# Research advances in the anti-inflammatory effects of SGLT inhibitors in type 2 diabetes mellitus

**DOI:** 10.1186/s13098-024-01325-9

**Published:** 2024-05-12

**Authors:** Ruining Zhang, Qingxing Xie, Xi Lu, Rongping Fan, Nanwei Tong

**Affiliations:** Department of Endocrinology, Center for Diabetes and Metabolism Research, West China Hospital of Sichuan University, Chengdu, China

**Keywords:** Sodium-glucose cotransporter inhibitors (SGLTis), Type 2 diabetes mellitus (T2D), Inflammation, Inflammatory biomarkers

## Abstract

Diabetes mellitus is one of the most significant global burden diseases. It is well established that a chronic, systemic, low-grade inflammatory condition is strongly correlated with type 2 diabetes mellitus (T2D) and the development of target-organ damage (TOD). Sodium-glucose cotransporter inhibitors (SGLTis), novel oral drugs for the treatment of diabetes, act mainly by reducing glucose reabsorption in proximal renal tubules and/or the intestine. Several high-quality clinical trials and large observational studies have revealed that SGLTis significantly improve cardiovascular and renal outcomes in T2D patients. Increasing evidence suggests that this is closely related to their anti-inflammatory properties, which are mainly manifested by a reduction in plasma concentrations of inflammatory biomarkers. This review analyses the potential mechanisms behind the anti-inflammatory effects of SGLTis in diabetes and presents recent evidence of their therapeutic efficacy in treating diabetes and related TOD.

**Graphic Abstract:**

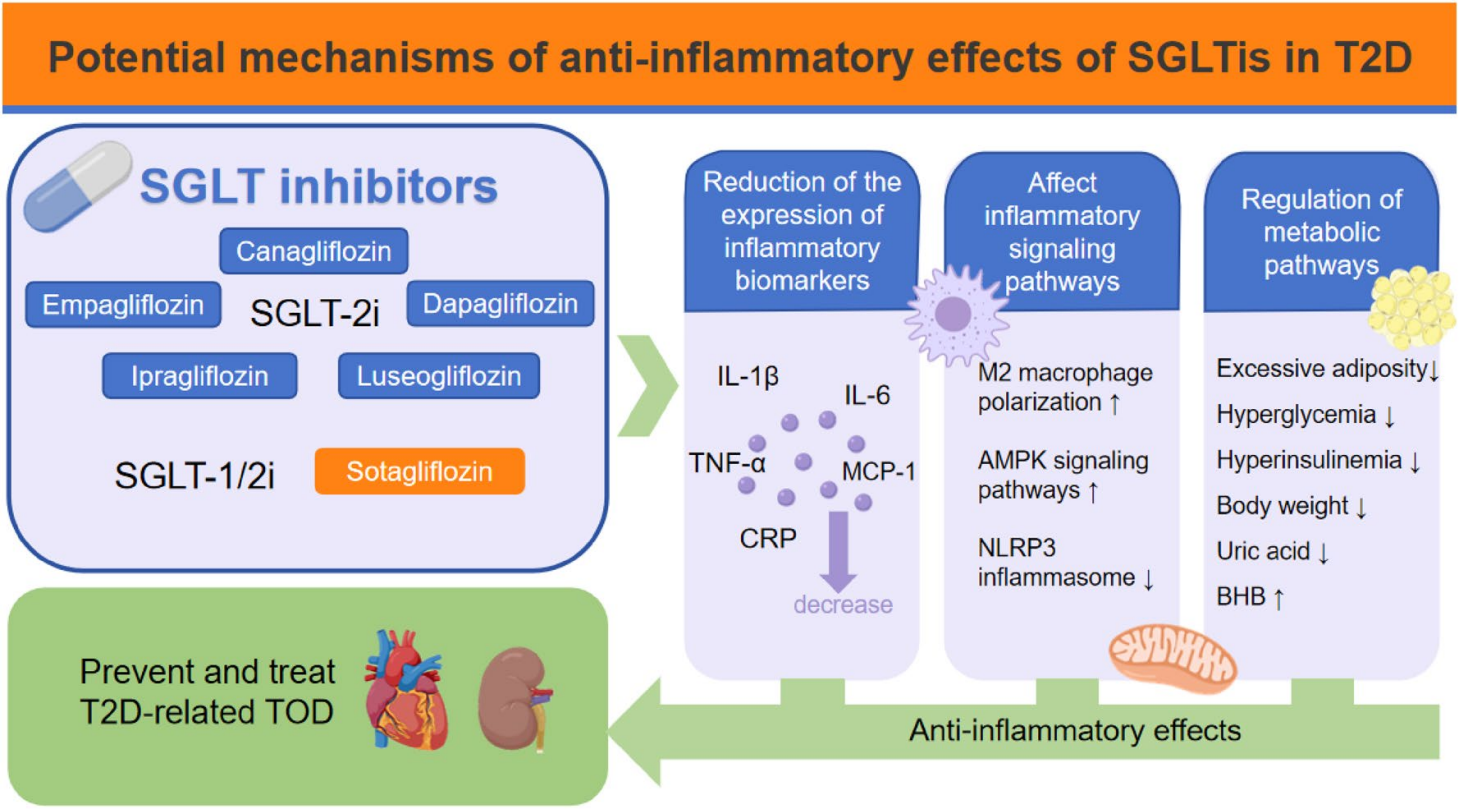

## Introduction

Type 2 diabetes mellitus (T2D) stands as a foremost ailment with substantial implications for human well-being. It has been recognized that a chronic, systemic, and low-grade inflammatory state is strongly combined with the development of T2D and target-organ damage (TOD) [1]. Plasma concentrations of inflammatory biomarkers, including but not limited to tumor necrosis factor (TNF)-a, interleukin (IL)-6, and C-reactive protein (CRP), are increased twofold in T2D [2]. Chronic tissue inflammation in the context of diabetes manifests within insulin-responsive tissues, notably including adipose tissue, the liver, skeletal muscle, and pancreatic islets. This phenomenon holds significant prominence as a pivotal etiological element in insulin resistance (IR) genesis and the subsequent onset of T2D. This chronic inflammatory state leads to long-term pathologic damage in diabetes, including metabolic dysfunction-associated steatotic liver disease (MASLD), retinopathy, cardiovascular disease (CVD), and nephropathy, and may also be responsible for the association of T2D with other diseases such as Alzheimer's disease, polycystic ovary syndrome (PCOS), gout, and rheumatoid arthritis (RA) [3]. In diabetes mellitus, a variety of factors are associated with the onset of severity in addition to hyperglycaemia, such as obesity, hyperuricaemia and persistently high levels of inflammatory biomarkers. The findings of the prestigious Canakinumab Anti-inflammatory Thrombosis Outcome Study (CANTOS) [4] confirmed that canakinumab targeting IL-1β reduces inflammation through the innate immune pathway, conferring direct cardiovascular (CV) benefits in CV high-risk populations. Consequently, there has been a growing emphasis on therapeutic modalities aimed at attenuating low-grade inflammation as a pivotal component of T2D management.

The utilization of SGLTis as novel antidiabetic drugs has significantly increased in medical practice over the last few years. SGLTis can reduce both fasting and postprandial hyperglycemia, preventing hyperglycemic toxicity. This is achieved by blocking glucose reabsorption in proximal renal tubules and/or intestine, increasing renal and/or intestinal excretion of glucose, and stimulating insulin secretion through its incretin and proliferator actions by binding to specific receptors in pancreatic β-cells [5, 6]. They have also been indicated to play an anti-inflammatory role in fat reduction, blood pressure lowering, efficacy in certain tumor diseases and, more recently, in cardiorenal protection [7, 8]. In fact, an increasing amount of fresh data suggests that SGLTis can inhibit inflammation, mainly in the form of reduced levels of relevant inflammatory biomarkers, including TNF-a, IL-1β, IL-6 and monocyte chemoattractant protein-1 (MCP-1) [9]. This review explores the possible ways in which SGLTis can reduce inflammation in patients with T2D. The mechanisms responsible for these anti-inflammatory effects are varied and include factors such as weight loss, reduced levels of uric acid, and decreased oxidative stress. Considering the central role that inflammation plays in the etiology and progression of diabetes, the principal objective of our comprehensive review is to systematically gather and analyse empirical substantiation pertaining to the anti-inflammatory properties of SGLTis, with particular emphasis on their application within the context of diabetic-associated inflammation. It also summarizes the benefits that SGLTis can offer to T2D patients.

## SGLT inhibition

### Pharmacological effect

**Fig. 1:**
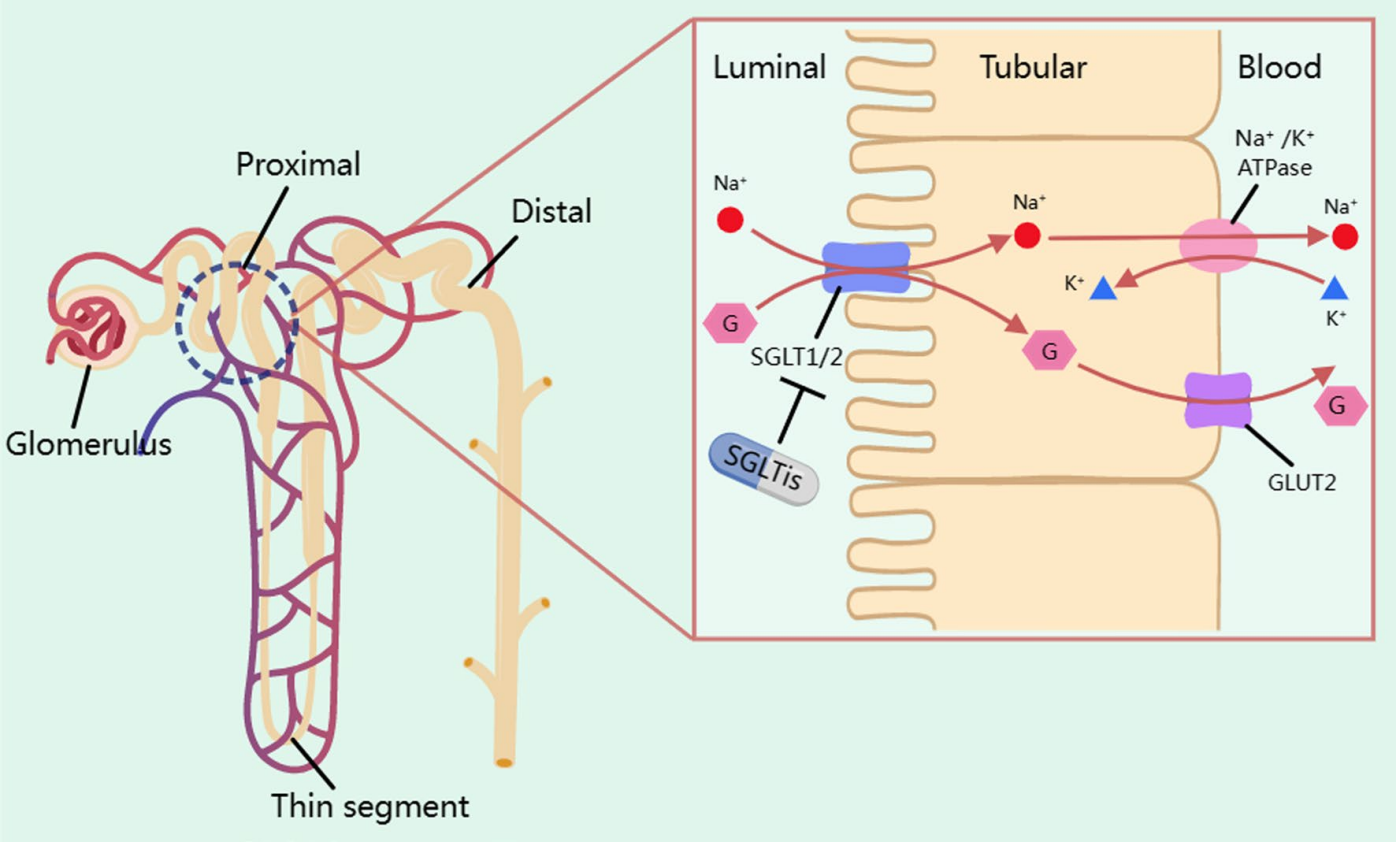
Mechanism of glucose-lowering activity of SGLTis in the kidneys. SGLT2 is located mainly in the S1 and S2 segments of the renal proximal tubule and SGLT1 is located mainly in the S3 segment of the renal proximal tubule. SGLTs, which are mainly located on the brush border (luminal side) of epithelial tubular cellis, enables the transfer of both sodium and glucose from the lumen into the tubular cells. Sodium is transported along with glucose through SGLTs. Next, glucose enters blood circulation through an active transport mechanism mediated by GLUT2, which are located on the basolateral membrane of the epithelial tubular cells. SGLTis reduces the reabsorption of glucose by inhibiting SGLT1 and SGLT2 targets in the kidneys, thereby allowing excess glucose to be excreted through the urine. GLUT2 is the major glucose transporter across the basolateral membrane

The SGLT gene family encompasses a minimum of six distinct isoforms within the human genome [10]. SGLT1 and SGLT2 have been extensively researched due to their vital role in transporting glucose and sodium through the intestines and renal cells. SGLT1 is responsible for glucose uptake in the small intestine and reabsorbs approximately 10% of the filtered glucose in renal proximal tubule segment 3 (S3), while SGLT2 is mostly found in renal proximal tubule S1-S2 and is responsible for reabsorbing approximately 90% of the filtered glucose load [11]. The combined effect of SGLT1 and SGLT2 results in less glucose in the urine. If SGLT1 and SGLT2 targets are inhibited, the glucose threshold is lowered by reducing glucose reabsorption in the small intestine and kidneys, thereby allowing excess glucose to be excreted through the urine (as depicted in Fig. 1).

Until now, the U.S. Food and Drug Administration (FDA) has approved canagliflozin, empagliflozin, dapagliflozin, ertugliflozin and sotagliflozin for treating T2D [12]. Sotagliflozin is the typical drug recognized as a dual-targeted sodium-glucose cotransporter 1/2 inhibitor (SGLT1/2i) which is currently available on the market. It is worth mentioning that the pharmacological action of canagliflozin is also related to SGLT1 inhibition in addition to SGLT2 inhibition, making it an important medication [13, 14].

### Evidence-based medicine for cardiorenal protective effects

Over the past few years, many clinical trials and observational studies have revealed that SGLTis can significantly reduce major adverse CV events (MACEs), hospitalization for heart failure (HF), CV-related and all-cause mortality, and renal outcomes [15–17]. Notably, non-T2D patients have also benefited from SGLTis, improving HF and kidney-related endpoints [18, 19]. In 2015, the Empagliflozin Cardiovascular Outcome Event Trial in Type 2 Diabetes Mellitus Patients (EMPA-REG OUTCOME) trial became the first study to address the CV outcomes of SGLTis in T2D patients at high CV risk. During the trial, 7020 patients received treatment for a median duration of 3.1 years. The findings showed that the Empagliflozin group exhibited a 38% decrease in mortality from cardiovascular causes, a 35% reduction in HF hospitalization, and a 32% decline in all-cause mortality compared to the placebo group [15]. Four subsequent large cardiovascular outcome trials (CVOTs) of SGLTis [Canagliflozin Cardiovascular Assessment Study (CANVAS), Dapagliflozin Effect on Cardiovascular Events-Thrombolysis In Myocardial Infarction 58 (DECLARE-TIMI 58), (Effect of Sotagliflozin on Cardiovascular Events in Patients with Type 2 Diabetes Post Worsening Heart Failure) SOLOIST-WHT, and (Effect of Sotagliflozin on Cardiovascular and Renal Events in Participants With Type 2 Diabetes and Moderate Renal Impairment Who Are at Cardiovascular Risk) SCORED] have exhibited a robust cardio-renal protective effect in a substantial cohort of patients diagnosed with T2D who are at elevated CV risk, in comparison to the placebo-administered group [16, 20–22]. In a meta-analysis undertaken by Zhang et al., which involved 351,476 patients suffering from T2D, it was found that SGLTi therapy resulted in substantial reductions in the risk of major adverse cardiac events, all-cause mortality, CV mortality, nonfatal MI, and hospitalization for HF [23]. Another meta-analysis of six SGLTis trials showcased a reduction in the primary atherosclerotic cardiovascular disease (ASCVD)-based composite of time to MACE. Therefore, SGLT2 inhibitors (empagliflozin, canagliflozin, dapagliflozin, ertugliflozin, or sotagliflozin) are recommended in the latest guidelines for patients with T2D who have a combination of multiple ASCVD risk factors or who have been diagnosed with ASCVD [24].

Furthermore, the Canagliflozin and Renal Events in Diabetes with Established Nephropathy Clinical Evaluation (CREDENCE) trial revealed groundbreaking results indicating for the first time that SGLTis can decelerate the advancement of chronic kidney disease (CKD) in diabetic individuals. This trial particularly observed the renal function of T2D patients who were administered canagliflozin, demonstrating a significant 30% decrease in the primary composite outcome of end-stage renal disease (ESRD) [25]. Another meta-analysis [26] included 11 CVOTs with data from 5 SGLTis (empagliflozin, canagliflozin, dapagliflozin, ertugliflozin, and sotagliflozin) and 77,541 participants, which demonstrated that treatment with SGLTis in patients with cardiometabolic disease and renal disease with SGLTis treatment moderately reduced combined CV death or HF hospitalization and significantly improved HF and CKD outcomes. In 2021, the SOLOIST-WHF trial revealed that sotagliflozin, an SGLT1/2i, significantly reduced the relative risk (RR) of the composite primary outcome (CV death, HF hospitalization, or HF emergency visit) by 33% compared with placebo (hazard ratio [HR] 0.67; 95% confidence interval [CI], 0.52–0.85) in individuals with diabetes and acute decompensated HF [21]. Subsequently, Dapagliflozin and Prevention of Adverse Outcomes in Heart Failure (DAPA-HF) and Empagliflozin Outcome Trial in Patients with Chronic Heart Failure with Reduced Ejection Fraction (EMPEROR-Reduced) and other studies further demonstrated the therapeutic effect of SGLTis. Specifically, both dapagliflozin and empagliflozin have been shown to decrease the occurrence of HF exacerbations and reduce the risk of CV mortality [19, 27]. Accordingly, in the latest guidelines, SGLT2 inhibitors (dapagliflozin, empagliflozin and sotagliflozin) are recommended for all patients with HFrEF and T2D to reduce the risk of HF hospitalization and CV mortality [24].

Extensive trials have provided strong evidence that SGLTis significantly decrease the risk of major CV events, HF hospitalizations, CV deaths, and renal outcomes. This is especially true for people with diabetes who have ASCVD, HF, and impaired renal function. Therefore, it is necessary to explore the mechanisms through which SGLTis provide significant protection to target organs.

## Anti-inflammatory evidence of SGLT inhibitors

As previously elucidated, inflammation assumes a pivotal role in the genesis and advancement of diabetes, consequently prompting a heightened scholarly interest in novel therapeutic approaches directed towards the mitigation of inflammation associated with diabetes. Evidence from a series of studies suggests that the anti-inflammatory effects of SGLTis may have an important effect on inhibiting the progression of diabetes and reducing the incidence of diabetic lesions and mortality.

Augmented concentrations of inflammatory biomarkers exhibit a correlative relationship with hyperglycemia and insulin dysregulation, thus bearing potential as predictive indicators for the onset of diabetes [28]. Numerous studies indicate that SGLTis can reduce the expression and release of inflammatory biomarkers such as IL-1β, IL-6, TNF-α, MCP-1, platelet endothelial cell adhesion molecule-1 (PECAM-1), vascular cell adhesion molecule-1 (VCAM-1), intercellular adhesion molecule-1 (ICAM-1) and CRP [29–32].

### Experimental evidence

#### Canagliflozin

A previous study demonstrated that canagliflozin exerted anti-inflammatory effects in lipopolysaccharide (LPS)-treated NIH mice. Canagliflozin (40 μM) significantly repressed the expression and release of TNF-α, IL-6, or IL-1 in LPS-induced RAW264.7 and THP-1 cells as well as in NIH mice, and these effects were shown to be mediated by inhibition of intracellular glycolysis, enhancement of autophagy and promotion of p62-mediated IL-1 degradation [33]. In a recent study, Western blotting of inflammatory tissues from the lungs of NIH mice showed that canagliflozin significantly reduced the protein levels of NOD-, LRR- and pyridine domain-containing protein 3 (NLRP3), IL-1β, and IL-18 and inhibited the phosphorylation of p65. Furthermore, qPCR tests revealed that canagliflozin caused changes in the mRNA levels of NLRP3, IL-1β, and IL-18 [34].

Another study showed that canagliflozin significantly reduced plasma concentrations of the inflammatory biomarkers IL-6, IL-1β and TNF-α in a dose-dependent manner in adenine-induced CKD rats. The concentrations and activities of inflammatory biomarkers correlated with the severity of CKD and renal function status, which may indicate that the beneficial effects of canagliflozin on the affected kidney are related to its anti-inflammatory effect [35]. During an experiment to study the impact of canagliflozin on MASLD in obese mice caused by a high-fat diet (HFD), it was found that canagliflozin was able to suppress the production of inflammatory biomarkers such as TNF-α, MCP-1, IL-1β, and IL-6. It also resulted in a reduction in the mRNA levels of these biomarkers in a dose-dependent manner in hepatocytes induced by a lipid mixture (LM) when compared to the effects of HFD administration alone [36]. The findings suggest that canagliflozin may delay the onset of MASLD by suppressing inflammation. In a separate study, male C57BL/6J mice fed with HFD and treated with canagliflozin for 8 weeks showed that, in addition to attenuating HFD-mediated increases in body weight and visceral and subcutaneous fat weights in mice, canagliflozin also demonstrated the capacity to mitigate the elevation in mRNA expression levels of the proinflammatory markers Iba1 and Il6. In addition, canagliflozin reduced the levels of inflammatory biomarkers and inhibited macrophage aggregation in the skeletal muscle of HFD-fed obese mice [37]. The findings suggest that canagliflozin treatment can not only stimulate caloric reduction but also inhibit obesity-related inflammation in the nervous system and skeletal muscle, thereby breaking the vicious cycle of obesity and inflammation.

#### Empagliflozin

UHPLC-MS/MS analysis showed an increased amount of branched-chain amino acids (BCAAs) in the retinas of diabetic mice. However, this accumulation could be inhibited by empagliflozin, especially at high doses. Empagliflozin protects the retina of db/db mice from inflammation, neovascularization and endothelial adhesion [38].

Empagliflozin (3 or 10 mg/kg/d) reduced plasma TNF-α levels and decreased the accumulation of M1-like macrophages while inducing an anti-inflammatory M2 phenotype in white adipose tissue (WAT) and intrahepatic macrophages and attenuating the chronic inflammation associated with obesity induced by a HFD in obese C57BL/6J mice [39]. Iannantuoni et al. found a significant decrease in hypersensitive C reactive protein (hs-CRP) levels in T2D patients after 24 weeks of treatment with empagliflozin [8].

Empagliflozin has been elucidated to possess anti-inflammatory attributes, as evidenced by its capacity to inhibit the proliferation of T-helper (Th) cells, diminish the presence of factors associated with Th17, and augment the functional characteristics of regulatory T cells (Tregs) [40]. Another study also demonstrated that empagliflozin ameliorated macrophage inflammation by inhibiting the expression of cyclooxygenase (COX)-2 and the release of prostaglandin E2 (PGE2) and iNOS genes in LPS-stimulated RAW 264.7 macrophages and by decreasing the secretion and mRNA expression of inflammatory biomarkers [TNF-α, IL-1β, IL-6, and interferon(IFR)-γ] [41].

#### Dapagliflozin

A cohort comprising male C57BL/6 wild-type and db/db mice, aged 12 weeks, was subjected to a 12-week intervention regimen, with either dapagliflozin administered at a dose of 1 mg/kg or an alternative drug. The outcomes of this investigation revealed that dapagliflozin intervention effectively attenuated the phosphorylation status of the NF-κBp65 subunit in db/db mice. Furthermore, the expression levels of COX-2, a downstream effector molecule subject to NF-κB regulation, exhibited a significant increase in the hepatic tissues of db/db mice. Nevertheless, dapagliflozin treatment yielded a substantial reduction in COX-2 expression levels in this context [42]. This study suggests that dapagliflozin treatment may alleviate metabolic dysfunction-associated steatohepatitis (MASH) by reducing the inflammatory response through inhibition of the NF-κB pathway.

Dapagliflozin (1.0 mg/kg/d) treatment of diabetic ApoE−/− mice for 12 weeks led to a reduction in the protein expression levels of NLRP3 and caspase-1 and an inhibition of the release of mature IL-1β and IL-18. Thus, dapagliflozin attenuated the activation of NLRP3 inflammatory vesicles, which in turn reduced the secretion of IL-1β and IL-18 in the livers of DM mice [43]. Another study also found that dapagliflozin attenuates the inflammatory response to myocardial ischaemia/reperfusion injury by reducing NLRP3 inflammatory vesicle-associated protein levels [44]. Streptozotocin (STZ)-induced DM rats showed significantly increased levels of inflammatory biomarkers in cardiac tissues of the DM group compared with the normal group. Following an 8 weeks of treatment with dapagliflozin (1.0 mg/kg/d), the cardiac tissues of rats belonging to the DM group exhibited a substantial reduction in the levels of IL-1β and IL-6. Additionally, there was an augmented immunostaining observed for NF-kB/p56 [45].

#### Luseogliflozin

According to a study, luseogliflozin has demonstrated the capacity to diminish the expression of inflammatory biomarkers induced by transient episodes of hyperglycemia. In nicotinamide and streptozotocin (NA/STZ)-treated ApoE KO mice, luseogliflozin normalized the expression of various genes related to inflammation, including F4/80, TNF-α, IL-6, IL-1β, PECAM-1,ICAM-1, matrix metalloproteinase(MMP)2, and MMP9 [29].

#### Ipragliflozin

Ipragliflozin (0.1–3 mg/kg/d) dramatically inhibited the increase in oxidative stress markers [protein carbonyls and thiobarbituric acid reactive substances (TBARS)] and inflammatory markers (TNF-a, IL-6, CRP, and MCP-1) in the plasma and liver of NA/STZ-induced diabetic mice in a dose-dependent manner [46]. The study suggests that ipragliflozin may improve hepatic steatosis in diabetic mice by reducing inflammation and oxidative stress.

#### Sotagliflozin

Diabetic patients are at higher risk of developing CV disease, and the SOLOIST-WHF trial, published in 2021, has shown that sotagliflozin has a significant CV benefit in diabetic patients with recent worsening of HF [21]. The cardiorenal protective effect is likely to be attributed to several potential mechanisms, and the anti-inflammatory effect of Sotagliflozin may be one of the important mechanisms. There is no experimental basis for studying the direct anti-inflammatory effects of Sotagliflozin, and further studies are needed.

### Clinical evidence

Numerous clinical trials were undertaken with the primary objective of scrutinizing the impact of SGLTis on pivotal inflammatory biomarkers. Within the framework of the Efficacy and Safety of Canagliflozin Versus Glimepiride in Patients with Type 2 Diabetes Inadequately Controlled with Metformin (CANTATA-SU) study, a post hoc exploratory analysis was undertaken to assess alterations in the levels of serum biomarkers, including leptin, adiponectin, IL-6, TNF-α, CRP, plasminogen activator inhibitor-1 (PAI-1), VCAM-1, and MCP-1, among patients with T2D over a 52-week duration relative to their baseline values. The results showed that by week 52, canagliflozin-treated T2D patients had a 25% decrease in median serum leptin and a 17% increase in median serum adiponectin compared to patients taking glimepiride. There was also a 22% reduction in median serum IL-6 and a slight trend towards a decrease in hs-CRP. These findings suggest that canagliflozin may improve adipose tissue function and have favorable effects on insulin sensitivity and CV disease risk [47]. Another prospective and open-label study enrolled 35 diabetic and stable chronic cardiac patients and revealed that canagliflozin treatment (100 mg/d for 12 months) resulted in a substantial decrease in hs-CRP compared to baseline (3 months: p = 0.002, 6 months: p = 0.001, 12 months: p = 0.007) [48].

Ninety-five patients with T2D and coronary heart disease (male 41.05%, mean age 62.85 ± 7.91 years, mean HbA1c 7.89 ± 0.96%) were randomized (1:1) to receive empagliflozin (10 mg/day) or placebo. After 26 weeks, individuals subjected to empagliflozin therapy exhibited reduced levels of IL-6 (− 1.06 pg/mL, 95% CI − 1.80 to − 0.32, P = 0.006), as well as lower levels of IL-1β and hs-CRP (− 4.58 pg/mL and − 2.86 mg/L; P-values 0.32 and 0.003, respectively) compared to those who received the placebo [49]. In a single-center, open-label, randomized, prospective study of 51 diabetic patients taking empagliflozin (10 mg/d for 12 months), hs-CRP levels in the blood were significantly reduced compared to baseline and placebo (− 74.4% compared to placebo and − 55.6% compared to baseline) [50]. In addition, in an observational prospective follow-up study evaluating the effects of empagliflozin on T2D inflammation, 15 diabetic patients taking empagliflozin (10 mg/d) showed a significant decrease in hs-CRP after 24 weeks of treatment and a significant increase in levels of the anti-inflammatory cytokine IL-10 after 24 weeks of treatment compared to baseline and placebo [8]. These studies provide some evidence for the anti-inflammatory properties of SGLTis in humans, which may contribute to their beneficial CV effects.

## Anti-inflammatory mechanisms of SGLTis

### Reduction of the expression of inflammatory biomarkers

T2D patients have increased plasma concentrations of inflammatory biomarkers such as TNF-α, IL-1β, IL-6, MCP-1, VCAM-1, ICAM-1 and PECAM-1, which are key components of the inflammatory signaling system [29–32]. Prolonged elevation of the levels of these molecules promotes IR in skeletal muscle as well as the release of acute-phase proteins such as CRP released by the liver [51]. Maintaining high levels of inflammatory biomarkers is robustly correlated with the progression of diabetes and has been implicated as a pivotal contributory factor in the pathogenesis of diabetic nephropathy, atherosclerosis and other diabetes-related TOD.

Much experimental evidence has shown that SGLTis can reduce the expression of circulating inflammatory biomarkers. Xu et al. demonstrated that canagliflozin (10–30 μM) exhibited a substantial reduction in mRNA expression of IL-1α and IL-6 and effectively restrained the release of TNF-α in LPS-treated RAW264.7 cells [33]. As previously delineated, in ApoE KO mice subjected to NA/STZ treatment, the administration of luseogliflozin resulted in a notable diminishment in the expression of inflammation-associated genes, encompassing TNF-α, IL-1β, IL-6, ICAM-1, PECAM-1, MMP2, and MMP9 [29].

### Affect inflammatory signaling pathways

#### Promoting M2 macrophage polarization

It is widely acknowledged that there are two distinct types of macrophages in the immune system: M1 and M2 macrophages. M1 macrophages produce cytokines including TNF-α, IL-1β and IL-6 which help sustain chronic inflammation. Conversely, M2 macrophages have anti-inflammatory and arterioprotective properties and release substances such as IL-1 receptor agonists, IL-10, and collagen [52]. In obese diabetic patients, adipose tissue macrophages (ATMs) play a key role in promoting inflammation and IR. This may extend to ectopic adipose tissue around the vasculature and heart, leading to CV pathology [53]. ATMs can be polarized into proinflammatory M1 macrophages and secrete several proinflammatory cytokines capable of disrupting insulin signaling, including TNF-a and IL-6, thereby mediating systemic chronic inflammation. Therefore, the differentiation of macrophages towards an anti-inflammatory phenotype can be considered a therapeutic strategy to ameliorate chronic inflammation in diabetic patients and inhibit the progression of diabetes and target-organ damage.

Several studies have shown that SGLTis promote M2 macrophage polarization, thereby alleviating inflammation. Xu et al. showed that empagliflozin increased M2 levels in WAT and decreased the release of M1-mediated inflammatory biomarkers in a mouse model fed a HFD [39]. Further studies have revealed that empagliflozin administration results in a decrease in M1 and an increase in M2. This indicates that empagliflozin promotes a shift towards an M2 macrophage phenotype and reduces T-cell accumulation in the WAT and liver, thereby mitigating obesity-induced IR and inflammation [54]. In addition, LPS-induced macrophages in humans and mice showed an increase in the number of M1 isoforms and the M1/M2 ratio, an effect that was completely reversed in a glucose-independent manner after dapagliflozin treatment [55]. Canagliflozin repeated the same results in LPS-induced mice and macrophages [56].

#### Inhibition of the NLRP3 inflammasome

The activation of NLRP3 inflammasome represents a pivotal molecular pathway involved in the orchestration of inflammation, serving as the conduit for the release of the proinflammatory biomarkers IL-1β and IL-18. This process assumes significant relevance in the pathogenesis of diabetic TOD [57]. Free fatty acids (FFA) and hyperglycemia have been demonstrated to activate the NLRP3 inflammasome in T2D [31].

Research investigations have revealed that SGLTis possess the capacity to impede the activation of the NLRP3 inflammasome. Presently, the suppressive impact of SGLTi therapy on NLRP3 inflammasome activation has been substantiated in both the heart and kidney [58]. The underlying mechanism entails the suppression of inflammasome initiation via calcium-dependent pathways, resulting in attenuated transcription levels of NLRP3, NF-kB, and caspase-1. In another study, Kim et al. illustrated that empagliflozin elicited elevations in serum beta-hydroxybutyric acid (BHB) concentrations and concomitantly decreased insulin levels among individuals afflicted with both T2D and CVD, without regard to their glycemic status [59]. Birnbaum et al. further elucidated that SGLTis exhibit inhibitory properties against NLRP3 inflammasome activation, thereby diminishing the mRNA expression levels of the proinflammatory cytokines IL-1β, IL-6, and TNF-α in BTBR ob/ob mice [60]. In murine models featuring STZ-induced diabetes in ApoE(−/−) mice and rodent models of T2D, empagliflozin exerts inhibitory effects on the IL-17A-induced secretion of IL-1β and IL-18 via modulation of the NLRP3/caspase1 signaling pathway. Moreover, the expression of IL-1β is reduced by inhibiting NF-κB phosphorylation/NLRP3 signaling in human macrophages [61]. Dapagliflozin has also been shown to inhibit IL-1β expression through NLRP3/caspase 1 signaling [59].

#### AMPK activation

Adenosine 5'-monophosphate-activated protein kinase (AMPK) is a serine/threonine kinase produced by activating energy pathways involved in regulating cell metabolism states [62, 63] and is the main regulator of cells and metabolism in the body. AMPK fosters the restoration of cellular energy equilibrium through the augmentation of adenosine triphosphate (ATP) synthesis and the concurrent reduction of ATP expenditure. This concerted action engenders an elevated adenosine diphosphate (ADP) ratio. The preservation of a heightened ATP/ADP ratio stands as a pivotal prerequisite for ensuring cellular viability [64].

Recent research findings have elucidated the participation of AMP-activated protein kinase (AMPK) in the realm of inflammation, with a particular focus on its role in the activation of the NLRP3 inflammasome. The anti-inflammatory attributes of AMPK have been empirically evidenced in individuals with diabetes under metformin treatment. This is associated with the inhibition of AMPK-mediated NLRP3 inflammasome activation and the subsequent release of IL-1β [65]. Previous studies have linked SGLTis to AMPK activation [58, 66, 67]. SGLTis have shown anti-inflammatory effects by activating AMPK signaling in diabetes models. Dapagliflozin can reduce NF-kB nuclear translocation in renal tubular human kidney-2 cells [68] and can also inhibit mice NLRP3 activation of inflammation of the body and the progression of diabetic nephropathy [60], which is mediated by AMPK. An additional investigation elucidated the anti-inflammatory properties of dapagliflozin in cardiac fibroblasts derived from T2D mice. Notably, these effects were ascribed to AMPK activation and occurred independently of SGLT1 involvement [58]. Canagliflozin induces AMPK activation in hepatocytes and HEK-293 cells, and concurrently, it exerts inhibition upon endothelial inflammatory biomarkers. This multifaceted mechanism involves both AMPK-dependent and AMPK-independent pathways [66]. In a clinical trial, the observed activation of AMPK at canagliflozin concentrations detected in human plasma was attributed to an elevation in intracellular AMP or ADP levels, stemming from the inhibition of mitochondrial respiratory chain [66]. In addition, another study further demonstrated that the activation of AMPK can blunt the inflammatory process and replenish energy levels [69].

The mechanism by which AMPK is activated to mediate anti-inflammatory effects may be due to the diminishment of glucose levels by SGLTis. In both LPS-stimulated in vitro and in vivo models, inflammatory biomarker levels were reduced [33, 69] and M2 macrophage expression was enhanced [69] after SGLTis were administered in an AMPK-dependent manner.

Furthermore, AMPK assumes a pivotal role in the modulation of autophagic processes and various other physiological functions [70]. Autophagy is governed by the intricate interplay of AMPK and the mammalian target of rapamycin (mTOR) signaling pathway [71]. In an experimental murine model of hepatic disease, empagliflozin effectively reinstated disrupted autophagy flux by eliciting AMPK activation and mTOR inhibition, concomitant with a noteworthy reduction in the release of IL-17 and IL-23 [72]. Dapagliflozin has additionally demonstrated the capacity to ameliorate autophagy impairments in rodent models afflicted with diabetes or obesity, where the predominant regulatory mechanism is the AMPK/mTOR signaling pathway [73].

### Regulation of metabolic pathways

#### Reduction of hyperglycemia

SGLT2i reduce the threshold of renal glucose excretion from approximately 10 mmol/L (180 mg/dL) to 2.2 mmol/L (40 mg/dL) [74]. The resultant elevation in urinary glucose excretion leads to a reduction in blood glucose levels. SGLT1i reduce the intestinal absorption of dietary glucose and increase the release of glucagon-like peptide-1, thereby improving glucose homeostasis [75]. Similar to other antihyperglycemic drugs, this drug can reduce hyperglycemic toxicity and improve the inflammation associated with hyperglycemia. Since the relationship between hyperglycemic toxicity and inflammation is classical, the mechanism is not detailed here.

#### Reduction in plasma insulin levels

The anti-inflammatory efficacy of SGLTis may be mechanistically linked to their capacity to attenuate insulin secretion. Experiments have shown that obesity-related hyperinsulinemia leads to inflammation of fat tissue in mice [76]. Hyperinsulinemia is postulated to be correlated with chronic low-grade inflammation in T2D patients, which is related to its level and duration [77, 78]. A recent investigation has demonstrated that individuals undergoing treatment with SGLTis exhibited a notable reduction in chronic low-grade inflammation, such as reduced levels of the important inflammatory biomarker IL-6, an effect that may be mediated by lower insulin levels. Insulin amplifies glucose uptake by macrophages, thereby reinforcing the proinflammatory state through the engagement of insulin receptors, modulation of glucose metabolism, and the generation of reactive oxygen species [79]. SGLTis reduce blood glucose levels due to its inhibition of reabsorption in the kidney, which can lead to a decrease in systemic insulin levels. Dapagliflozin, a pharmacological agent known for its capacity to diminish hyperinsulinemia while promoting weight loss, has demonstrated the ability to augment insulin clearance, consequently leading to a further decline in systemic insulin concentrations [80].

#### Increase in beta-hydroxybutyrate levels

A salient attribute discerned in individuals afflicted by diabetes receiving therapeutic intervention involving SGLTis is the discernible augmentation in the presence of circulating ketone bodies within their physiological milieu [81]. Ferannini et al. postulated that the glycosuria induced by empagliflozin results in a reduced insulin-to-glucagon ratio, thereby promoting increased hepatic FFA oxidation, consequently leading to an upsurge in circulating BHB levels [82]. In diabetic patients, SGLTis can increase BHB levels up to 0.6 mmol/L [83]. BHB may demonstrate anti-inflammatory attributes via its capacity to inhibit the NLRP3 inflammasome, thereby resulting in a consequential diminishment in the synthesis of inflammatory biomarkers [84]. Additionally, BHB may serve as an epigenetic modifier. Multiple investigations have underscored the regulatory influence of BHB on the NLRP3 inflammasome. Youm et al. were the pioneering researchers to elucidate the influence of BHB on the NLRP3 inflammasome and its concomitant IL-1β secretion in both human macrophages and murine models [85]. Subsequently, Byrne et al. demonstrated that high BHB inhibits NLRP3 inflammasome activation in HF mice, reducing inflammatory biomarkers and macrophage infiltration in cardiac tissue [86]. Another study has shown that BHB serves as an endogenous and selective inhibitor of Class I histone deacetylases (HDACs), and high BHB can reduce various inflammatory biomarkers by inhibiting histone pro-acetylases [81]. In addition to these anti-inflammatory mechanisms, direct epigenetic modification of adiponectin genomic protein H3K9 by BHB is associated with a decrease in proinflammatory biomarkers in 3T3-L1 adipocytes [87]. Reports supporting the hormetic action of ketone bodies have shown that SGLTi therapy can induce the activity of Nrf2, AMPK, and sirtuins, along with downregulation of the NLRP3 inflammasome, thus providing significant cardiac protection [88]. Hence, the suppression of the NLRP3 inflammasome and HDACs, or the augmentation of adiponectin expression via the elevation of BHB, may constitute contributory factors to the anti-inflammatory mechanisms associated with SGLTis. Increased ketone body production during SGLTi treatment is considered to be a mechanism to prevent CV death and HF. On the other hand, it has been shown that largely increased ketone body concentrations may contribute to the development of diabetic ketoacidosis [81].

#### Body weight and fat reduction

SGLTis have the potential to mitigate surplus adipose tissue, a condition highly correlated with chronic low-grade inflammation. The mechanism by which SGLTis cause loss of adipose tissue cannot simply be explained by the caloric loss caused by diabetes. Studies have shown that in T2D patients, the diminished insulin-to-glucagon ratio, instigated by the attenuation of plasma glucose levels through the administration of SGLTis, can redirect energy metabolism towards increased utilization of fat, augmented fatty acid oxidation, and heightened ketogenesis [53].

Excessive adiposity in individuals with T2D is marked by the pronounced enlargement of WAT in the context of escalated energy storage demands. This expansion is coupled with compromised angiogenic processes, infiltration of macrophages, and a heightened state of inflammation attributed to hypoxia and the apoptotic demise of adipocytes [89]. Consequently, the process of adipose tissue differentiation becomes compromised, predisposing individuals to the accumulation of ectopic fat within organs such as the heart and vasculature. In the context of obesity, perivascular adipose tissue and epicardial adipose tissue accumulate and release a spectrum of proinflammatory mediators, such as leptin, resistin, and various adipokines, while the production of adiponectin experiences a decline [90]. A post hoc analysis of a clinical trail involving canagliflozin and glimepiride revealed that canagliflozin induced a 25% decrease in serum leptin levels, accompanied by a 17% elevation in the concentrations of adiponectin, an anti-inflammatory adipokine, in comparison to the effects observed with glimepiride [47]. SGLTis-induced volume reduction and inflammation of perivisceral adipose tissue may ameliorate cardiac fibrosis and glomerulosclerosis by reducing paracrine inflammatory effects on visceral organs, including the heart and kidneys [91]. Significantly, SGLTis exhibit a pharmacological capacity to diminish epicardial adipose tissue, a phenomenon linked to the mitigation of inflammation and the suppression of the secretion of detrimental adipokines. This effect can have a favorable impact on the structural and functional aspects of the adjacent myocardium [92]. A research study was conducted to assess and compare the impact of dapagliflozin in comparison to standard coronary treatment on the volume of epicardial adipose tissue in a cohort comprising 40 individuals diagnosed with T2D concomitant with coronary artery disease. Following a 6-month intervention period, the group subjected to dapagliflozin exhibited a notable decline in epicardial adipose tissue volume, concomitant with a reduction in levels of TNF-a and PAI-1, compared with the conventional treatment group [93]. Numerous meta-analytic investigations encompassing clinical trials involving patients diagnosed with T2D have consistently demonstrated substantial reductions in body weight subsequent to the administration of SGLTis [94, 95]. This phenomenon could potentially be associated with heightened fat utilization, reduced adipose tissue volume, and the conversion of white adipose tissue to a more metabolically active state, thereby exacerbating the attenuation of IR induced by obesity [96–100]. A recent study showed that empagliflozin resulted in significant weight loss in elderly T2D patients with BMI ≥ 22 kg/m^2^, primarily by reducing fat mass rather than muscle mass, without affecting muscle mass or strength [101].

#### Reduction in plasma uric acid

Hyperuricaemia (HUA) is commonly seen in T2D patients due to impaired renal excretion of uric acid (UA) associated with IR and hyperinsulinaemia [102]. Uric acid crystals can induce the secretion of the proinflammatory cytokine IL-1β and activate the NLRP3 inflammasome in macrophages and immune cells [103]. A study that stimulated macrophages with UA showed that even the soluble form of UA can activate this pathway [103]. Uric acid crystals additionally serve as pro-oxidant entities, diminishing the bioavailability of nitric oxide, amplifying the generation of reactive oxygen species (ROS), and eliciting activation of the NF-κB and MAPK signaling pathways [104].UA levels are positively correlated with many inflammatory biomarkers [105].

The EMPA-REG OUTCOME study [15] showed that treatment with SGLTis can reduce circulating UA levels. A subanalysis of the study investigated the correlation between plasma UA levels and cardiac as well as renal outcomes in patients with T2D. According to the analysis, there seems to be a link between lower levels of plasma UA and a decrease in HF hospitalizations and CV mortality [106]. The latest study collected observational data on 15,067 adults diagnosed with gout and T2D between 2014 and 2020. In the investigation, individuals who commenced treatment with SGLTis exhibited a notably reduced recurrence rate in comparison to those who initiated therapy with dipeptidyl peptidase-4 (DPP-4) inhibitors, quantified at 52.4 and 79.7 per 1000 person-years, respectively. Correspondingly, the RR and rate difference (RD) for instances of gout-related primary emergency department visits and hospitalizations were 0.52 (CI 0.32 to 0.84) and − 3.4 (CI − 5.8 to − 0.9) per 1000 person-years, respectively. This study demonstrates that SGLTis can provide significant benefits for gout in T2D patients with gout [107].

### Inhibition of oxidative stress

Strictly related to their anti-inflammatory properties, SGLTis can also correct oxidative stress [108]. At present, a body of empirical research has substantiated the impact of SGLTis on oxidative stress. In diabetic rodent models, ipragliflozin reduced the levels of oxidative stress biomarkers (TBARS and protein carbonyls) in the plasma and liver. Empagliflozin exerts the same effect in rat models of T1D and T2D [109]. Furthermore, diabetic patients under treatment with canagliflozin displayed heightened expression of SIRT-6 within atherosclerotic plaques, along with diminished indicators of oxidative stress and inflammation [110].

Numerous studies have shown that excess ROS can lead to activation of the NLRP3 inflammasome [111–113]. An experimental study on dapagliflozin in experimental steatohepatitis observed that dapagliflozin significantly reduced ROS production in the liver tissues of diabetic and nondiabetic mice. Subsequent examinations revealed that diabetic mice subjected to dapagliflozin treatment exhibited reduced protein expression levels of NLRP3 and caspase-1, concomitant with suppressed release of mature IL-1β and IL-18. Furthermore, dapagliflozin administration led to ameliorated steatohepatitis in diabetic mice involved in an experimental steatohepatitis study, with this improvement being connected with the attenuation of hepatic ROS and the modulation of NLRP3 inflammasome activation by dapagliflozin [43]. It has been reported that SGLTi can reduce the inflammatory response in the kidneys of diabetic patients by decreasing the expression of MCP-1, p65, Toll-like receptor 4 and osteopontin. This suggests that SGLTis may reduce oxidative damage in part by inhibiting inflammatory responses in several pathways [108].

## Discussion

**Fig. 2:**
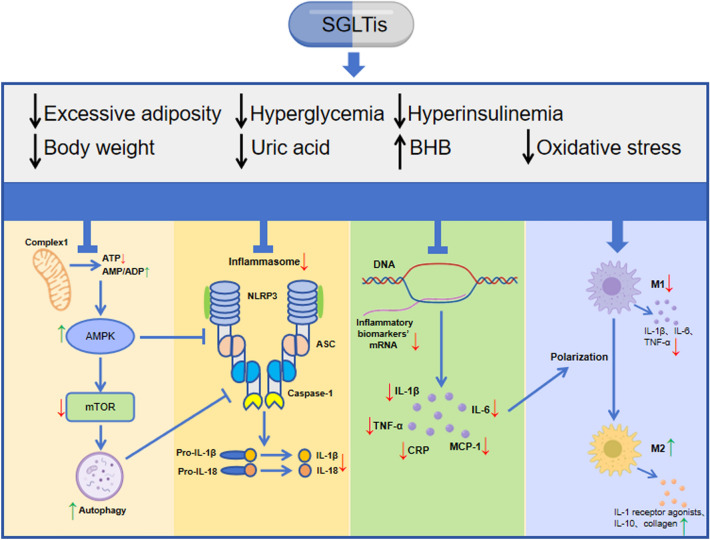
Mechanisms of the anti-inflammatory effects of SGLTis in diabetes. SGLTis can effectively reduce the levels of inflammatory markers (including but not limited to TNF-α, IL-1β, IL-6, MCP-1, and CRP), and affect the inflammatory signaling pathways by activating AMPK, inhibiting the activation of NLRP3 inflammasome, and promoting the polarization of M2 macrophages, thereby directly inhibiting diabetic inflammation. In addition, SGLTis also exert indirect anti-inflammatory effects by regulating various metabolic pathways, such as reducing hyperglycemia, hyperinsulinemia, uric acid levels, body weight and excessive adipose tissue, and increasing ketone body levels. There is a close interaction between inflammation and oxidative stress, and the reduction of oxidative stress levels by SGLTis can alleviate the inflammatory process. SGLTis, Sodium-glucose cotransporter inhibitors; ATP, adenosine triphosphate; ADP, adenosine diphosphate; AMP, adenosine monophosphate; AMPK, adenosine 5'-monophosphate-activated protein kinase; mTOR, mechanistic target of rapamycin; NLRP3, NOD-, LRR- and pyridine domain-containing protein 3; IL, Interleukin; TNF-α, Tumour necrosis factor-α; MCP-1, Monocyte chemoattractant protein-1; CRP, C-reactive protein; Blue arrows, represent the normal signalling pathway;↑, increase;↓, decrease

T2D is strongly associated with comorbidities such as obesity, MASLD, hypertension and dyslipidemia, as well as with a variety of hyperglycemia-related TOD such as CKD and HF. As a result, there is a constant need for new drugs that can manage hyperglycemia and help with the treatment of T2D-related TOD and comorbidities. SGLTis, a novel oral antidiabetic agent, has revolutionized the comprehensive management of diabetic patients. SGLTis have a wider role beyond controlling blood glucose. A succession of sizable clinical investigations has demonstrated that SGLTis can engender a noteworthy cardiorenal protective effect, potentially through their anti-inflammatory attributes, which appear to operate, at least in part, in a manner dissociated from blood glucose regulation. This intriguing observation underscores the potential efficacy of SGLTis as a therapeutic approach in mitigating cardiorenal complications in diabetic patients. A mounting body of evidence derived from diverse in vitro and in vivo investigations indicates that SGLTis possess the capacity to effectively attenuate the release of inflammatory markers and the mRNA expression of proinflammatory cytokines, including but not limited to TNF-α, IL-1β, IL-6, and MCP-1. They also affect the inflammatory signaling pathway by activating AMPK, inhibiting the activation of the NLRP3 inflammasome, and promoting the polarization of M2 macrophages, thereby having a direct inhibitory effect on the relevant inflammatory signaling system and overall anti-inflammatory effect. Regarding other anti-inflammatory mechanisms, SGLTis act as an anti-inflammatory effect indirectly by modulating metabolic pathways, including lowering adipose tissue inflammation, uric acid levels, postprandial hyperglycemia and hyperinsulinemia, as well as elevating ketone bodies (as depicted in Fig. 2).

There is a lack of relevant research evidence on the anti-inflammatory effect of the SGLT1/2i sotagliflozin, which is estimated to have the strongest anti-inflammatory effect of SGLTis. This is because it inhibits not only SGLT2 but also SGLT1, which has a strong effect on the gut and may have positive effects on gut bacteria and hormones. This could be why it is the only SGLTis that can reduce macrovascular events in patients with T2D [21]. There is no disagreement that inflammation causes atherosclerosis.

Nonetheless, contemporary investigations into the impact of SGLTis on inflammation within cohorts afflicted by associated disorders exhibit certain limitations, notably the constraints imposed by small-scale sample sizes. Significantly, additional clinical trials are imperative to establish the causal relationship between these anti-inflammatory effects and the observed reductions in the incidence of MACE, HF, CV mortality, and the risk of renal outcomes, as evidenced in recent randomized controlled trials employing SGLTis. In addition, it is important to assess inflammatory biomarkers in specific organs and tissues of diabetic patients using SGLTis. This will help determine how SGLTis can directly contribute to reducing diabetes-related TOD and mortality. We are eagerly waiting for more findings to explore the molecular mechanisms associated with the anti-inflammatory properties of SGLTis, which could be beneficial in developing new drugs for the prevention and management of T2D-related TOD.

## Data Availability

Not applicable.

## Abbreviations

T2D: Type 2 diabetes mellitus
TOD: Target-organ damage
SGLTis: Sodium-glucose cotransporter inhibitors
TNF: Tumour necrosis factor
IL: Interleukin
CRP: C-reactive protein
IR: Insulin resistance
MASLD: Metabolic dysfunction-associated steatotic liver disease
CVD: Cardiovascular disease
PCOS: Polycystic ovary syndrome
RA: Rheumatoid arthritis
CANTOS: Canakinumab Anti-inflammatory Thrombosis Outcome Study
CV: Cardiovascular
MCP-1: Monocyte chemoattractant protein-1
S3: Segment 3
FDA: Food and Drug Administration
MACE: Major adverse CV events
SGLT1/2i: Sodium-glucose cotransporter 1/2 inhibitor
HF: Heart failure
EMPA-REG OUTCOME: Empagliflozin Cardiovascular Outcome Event Trial in Type 2 Diabetes Mellitus Patients
CVOTs: Cardiovascular Outcome Trials Assessment Study
CANVAS: Canagliflozin Cardiovascular Assessment Study
DECLARE-TIMI 58: Dapagliflozin Effect on Cardiovascular Events-Thrombolysis In Myocardial Infarction 58
SOLOIST-WHF: Effect of Sotagliflozin on Cardiovascular Events in Patients with Type 2 Diabetes Post Worsening Heart Failure
SCORED: Effect of Sotagliflozin on Cardiovascular and Renal Events in Participants With Type 2 Diabetes and Moderate Renal Impairment Who Are at Cardiovascular Risk
ASCVD: Atherosclerotic cardiovascular disease
CREDENCE: Canagliflozin and Renal Events in Diabetes with Established Nephropathy Clinical Evaluation
CKD: Chronic kidney disease
ESRD: End-stage renal disease
RR: Relative risk
HR: Hazard ratio
CI: Confidence interval
DAPA-HF: Dapagliflozin and Prevention of Adverse Outcomes in Heart Failure
EMPEROR-Reduced: Empagliflozin Outcome Trial in Patients with Chronic Heart Failure with Reduced Ejection Fraction
PECAM-1: Platelet endothelial cell adhesion molecule-1
VCAM-1: Vascular cell adhesion molecule-1
ICAM-1: Intercellular adhesion molecule-1
LPS: Lipopolysaccharide
NLRP3: NOD-, LRR- and pyridine domain-containing protein 3
HFD: High-fat diet
LM: Lipid mixture
BCAAs: Branched-chain amino acids
WAT: White adipose tissue
hs-CRP: Hypersensitive C reactive protein
Th: T-helper
Tregs: Regulatory T cells
COX: Cyclooxygenase
PGE2: Prostaglandin E2
IFR: Interferon
MASH: Metabolic dysfunction-associated steatohepatitis
STZ: Streptozotocin
NA/STZ: Nicotinamide and streptozotocin
MMP: Matrix metalloproteinase
TBARS: Thiobarbituric acid reactive substances
CANTATA-SU: Efficacy and safety of canagliflozin versus glimepiride in patients with type 2 diabetes inadequately controlled with metformin
PAI-1: Plasminogen activator inhibitor-1
ATMs: Adipose tissue macrophages
FFA: Free fatty acids
BHB: Beta-hydroxybutyric acid
AMPK: Adenosine 5'-monophosphate-activated protein kinase
ATP: Adenosine triphosphate
ADP: Adenosine diphosphate
mTOR: Mammalian target of rapamycin
HDACs: Histone deacetylases
HUA: Hyperuricemia
UA: Uric acid
ROS: Reactive oxygen species
DPP-4: Dipeptidyl peptidase-4
RD: Rate difference

## References

[1] Prattichizzo F, De Nigris V, Spiga R (2018). Inflammageing and metaflammation: the yin and yang of type 2 diabetes. Ageing Res Rev.

[2] Petersen AMW, Pedersen BK (2005). The anti-inflammatory effect of exercise. J Appl Physiol.

[3] Rohm TV, Meier DT, Olefsky JM (2022). Inflammation in obesity, diabetes and related disorders. Immunity.

[4] Ridker PM, Everett BM, Thuren T (2017). Antiinflammatory Therapy with Canakinumab for Atherosclerotic Disease[J]. N Engl J Med.

[5] Kramer CK, Zinman B (2019). Sodium-glucose cotransporter-2 (SGLT-2) inhibitors and the treatment of type 2 diabetes. Annu Rev Med.

[6] Fonseca VA (2014). New developments in diabetes management: medications of the 21st century. Clin Ther.

[7] Elnaem MH, Mansour NO, Nahas AF (2020). Renal outcomes associated with the use of non-insulin antidiabetic pharmacotherapy: a review of current evidence and recommendations. Int J General Med.

[8] Iannantuoni F, de Marañon AM, Diaz-Morales N (2019). The SGLT2 inhibitor empagliflozin ameliorates the inflammatory profile in type 2 diabetic patients and promotes an antioxidant response in leukocytes. J Clin Med.

[9] Scisciola L, Cataldo V, Taktaz F (2022). Anti-inflammatory role of SGLT2 inhibitors as part of their anti-atherosclerotic activity: data from basic science and clinical trials. Front Cardiovasc Med..

[10] Scheepers A, Joost H-G, Schürmann A (2004). The glucose transporter families SGLT and GLUT: molecular basis of normal and aberrant function. J Parenter Enteral Nutr.

[11] Wright EM, Loo DDF, Hirayama BA (2011). Biology of human sodium glucose transporters. Physiol Rev.

[12] Ci C (2016). Sodium-glucose cotransporter 2 (SGLT2) inhibitors from natural products: discovery of next-generation antihyperglycemic agents. Molecules.

[13] Sokolov V, Yakovleva T, Chu L (2020). Differentiating the sodium-glucose cotransporter 1 inhibition capacity of canagliflozin vs. dapagliflozin and empagliflozin using quantitative systems pharmacology modeling. CPT Pharmacometr Syst Pharmacol.

[14] Dasari D, Goyal SG, Penmetsa A (2023). Canagliflozin protects diabetic cardiomyopathy by mitigating fibrosis and preserving the myocardial integrity with improved mitochondrial function. Eur J Pharmacol.

[15] Zinman B, Wanner C, Lachin JM (2015). Empagliflozin, cardiovascular outcomes, and mortality in type 2 diabetes. N Engl J Med.

[16] Neal B, Perkovic V, Mahaffey KW (2017). Canagliflozin and cardiovascular and renal events in type 2 diabetes. N Engl J Med.

[17] Li C-X, Liang S, Gao L (2021). Cardiovascular outcomes associated with SGLT-2 inhibitors versus other glucose-lowering drugs in patients with type 2 diabetes: a real-world systematic review and meta-analysis. PLoS ONE.

[18] Heerspink HJL, Stefánsson BV, Correa-Rotter R (2020). Dapagliflozin in patients with chronic kidney disease. N Engl J Med.

[19] McMurray JJV, Solomon SD, Inzucchi SE (2019). Dapagliflozin in patients with heart failure and reduced ejection fraction. N Engl J Med.

[20] Wiviott SD, Raz I, Bonaca MP (2019). Dapagliflozin and cardiovascular outcomes in type 2 diabetes. N Engl J Med.

[21] Bhatt DL, Szarek M, Steg PG (2021). Sotagliflozin in patients with diabetes and recent worsening heart failure. N Engl J Med.

[22] Bhatt DL, Szarek M, Pitt B (2021). Sotagliflozin in patients with diabetes and chronic kidney disease. N Engl J Med.

[23] Zhang X-L, Zhu Q-Q, Chen Y-H (2018). Cardiovascular safety, long-term noncardiovascular safety, and efficacy of sodium-glucose cotransporter 2 inhibitors in patients with type 2 diabetes mellitus: a systemic review and meta-analysis with trial sequential analysis. J Am Heart Assoc.

[24] Marx N, Federici M, Schütt K (2023). 2023 ESC Guidelines for the management of cardiovascular disease in patients with diabetes. Eur Heart J.

[25] Perkovic V, Jardine MJ, Neal B (2019). Canagliflozin and renal outcomes in type 2 diabetes and nephropathy. N Engl J Med.

[26] Giugliano D, Longo M, Scappaticcio L (2021). SGLT-2 inhibitors and cardiorenal outcomes in patients with or without type 2 diabetes: a meta-analysis of 11 CVOTs. Cardiovasc Diabetol.

[27] Packer M, Anker SD, Butler J (2020). Cardiovascular and renal outcomes with empagliflozin in heart failure. N Engl J Med.

[28] Pradhan AD, Manson JE, Rifai N (2001). C-reactive protein, interleukin 6, and risk of developing type 2 diabetes mellitus. JAMA.

[29] Nakatsu Y, Kokubo H, Bumdelger B (2017). The SGLT2 inhibitor luseogliflozin rapidly normalizes aortic mRNA levels of inflammation-related but not lipid-metabolism-related genes and suppresses atherosclerosis in diabetic ApoE KO mice. Int J Mol Sci.

[30] Dimitriadis GK, Nasiri-Ansari N, Agrogiannis G (2019). Empagliflozin improves primary haemodynamic parameters and attenuates the development of atherosclerosis in high fat diet fed APOE knockout mice. Mol Cell Endocrinol.

[31] Leng W, Ouyang X, Lei X (2016). The SGLT-2 inhibitor dapagliflozin has a therapeutic effect on atherosclerosis in diabetic ApoE-/- Mice. Mediat Inflamm.

[32] Lee DM, Battson ML, Jarrell DK (2018). SGLT2 inhibition via dapagliflozin improves generalized vascular dysfunction and alters the gut microbiota in type 2 diabetic mice. Cardiovasc Diabetol.

[33] Xu C, Wang W, Zhong J (2018). Canagliflozin exerts anti-inflammatory effects by inhibiting intracellular glucose metabolism and promoting autophagy in immune cells. Biochem Pharmacol.

[34] Niu Y, Zhang Y, Zhang W (2022). Canagliflozin ameliorates NLRP3 inflammasome-mediated inflammation through inhibiting NF-κB signaling and upregulating Bif-1. Front Pharmacol.

[35] Ali BH, Al Salam S, Al Suleimani Y (2019). Effects of the SGLT-2 inhibitor canagliflozin on adenine-induced chronic kidney disease in rats. Cell Physiol Biochem.

[36] Xu Z, Hu W, Wang B (2022). Canagliflozin ameliorates nonalcoholic fatty liver disease by regulating lipid metabolism and inhibiting inflammation through induction of autophagy. Yonsei Medical J.

[37] Naznin F, Sakoda H, Okada T (2017). Canagliflozin, a sodium glucose cotransporter 2 inhibitor, attenuates obesity-induced inflammation in the nodose ganglion, hypothalamus, and skeletal muscle of mice. Eur J Pharmacol.

[38] Gong Q, Zhang R, Wei F (2022). SGLT2 inhibitor-empagliflozin treatment ameliorates diabetic retinopathy manifestations and exerts protective effects associated with augmenting branched chain amino acids catabolism and transportation in db/db mice. Biomed Pharmacother.

[39] Xu L, Nagata N, Nagashimada M (2017). SGLT2 inhibition by empagliflozin promotes fat utilization and browning and attenuates inflammation and insulin resistance by polarizing M2 macrophages in diet-induced obese mice. EBioMedicine.

[40] Borzouei S, Moghimi H, Zamani A (2021). Changes in T helper cell-related factors in patients with type 2 diabetes mellitus after empagliflozin therapy. Hum Immunol.

[41] Lee N, Heo YJ, Choi S-E (2021). Anti-inflammatory effects of empagliflozin and gemigliptin on LPS-stimulated macrophage via the IKK/NF-κB, MKK7/JNK, and JAK2/STAT1 signalling pathways. J Immunol Res.

[42] Qiao P, Jia Y, Ma A (2022). Dapagliflozin protects against nonalcoholic steatohepatitis in db/db mice. Front Pharmacol.

[43] Leng W, Wu M, Pan H (2019). The SGLT2 inhibitor dapagliflozin attenuates the activity of ROS-NLRP3 inflammasome axis in steatohepatitis with diabetes mellitus. Ann Transl Med.

[44] Yu Y-W, Que J-Q, Liu S (2021). Sodium-glucose co-transporter-2 inhibitor of dapagliflozin attenuates myocardial ischemia/reperfusion injury by limiting NLRP3 inflammasome activation and modulating autophagy. Front Cardiovasc Med.

[45] El-Shafey M, El-Agawy MSE-D, Eldosoky M (2022). Role of dapagliflozin and liraglutide on diabetes-induced cardiomyopathy in rats: implication of oxidative stress, inflammation, and apoptosis. Front Endocrinol.

[46] Tahara A, Takasu T (2020). Therapeutic effects of SGLT2 inhibitor ipragliflozin and metformin on NASH in type 2 diabetic mice. Endocrine Res.

[47] Garvey WT, Van Gaal L, Leiter LA (2018). Effects of canagliflozin versus glimepiride on adipokines and inflammatory biomarkers in type 2 diabetes. Metabolism.

[48] Real J, Vlacho B, Ortega E (2021). Cardiovascular and mortality benefits of sodium-glucose co-transporter-2 inhibitors in patients with type 2 diabetes mellitus: CVD-Real Catalonia. Cardiovasc Diabetol.

[49] Gohari S, Reshadmanesh T, Khodabandehloo H (2022). The effect of EMPAgliflozin on markers of inflammation in patients with concomitant type 2 diabetes mellitus and Coronary ARtery Disease: the EMPA-CARD randomized controlled trial. Diabetol Metab Syndr.

[50] Hattori S (2018). Anti-inflammatory effects of empagliflozin in patients with type 2 diabetes and insulin resistance. Diabetol Metab Syndr.

[51] Calle MC, Fernandez ML (2012). Inflammation and type 2 diabetes. Diabetes Metab.

[52] Barrett TJ (2020). Macrophages in atherosclerosis regression. Arterioscler Thromb Vasc Biol.

[53] Elrakaybi A, Laubner K, Zhou Q (2022). Cardiovascular protection by SGLT2 inhibitors - Do anti-inflammatory mechanisms play a role?. Mol Metab.

[54] Xu L, Nagata N, Chen G (2019). Empagliflozin reverses obesity and insulin resistance through fat browning and alternative macrophage activation in mice fed a high-fat diet. BMJ Open Diabetes Res Care.

[55] Balzer MS, Rong S, Nordlohne J (2020). SGLT2 inhibition by intraperitoneal dapagliflozin mitigates peritoneal fibrosis and ultrafiltration failure in a mouse model of chronic peritoneal exposure to high-glucose dialysate. Biomolecules.

[56] Lin F, Song C, Zeng Y (2020). Canagliflozin alleviates LPS-induced acute lung injury by modulating alveolar macrophage polarization. Int Immunopharmacol.

[57] Pawlos A, Broncel M, Woźniak E (2021). Neuroprotective effect of SGLT2 inhibitors. Molecules.

[58] Ye Y, Bajaj M, Yang H-C (2017). SGLT-2 inhibition with dapagliflozin reduces the activation of the Nlrp3/ASC inflammasome and attenuates the development of diabetic cardiomyopathy in mice with type 2 diabetes. Further augmentation of the effects with saxagliptin, a DPP4 inhibitor. Cardiovasc Drugs Ther.

[59] Kim SR, Lee S-G, Kim SH (2020). SGLT2 inhibition modulates NLRP3 inflammasome activity via ketones and insulin in diabetes with cardiovascular disease. Nat Commun.

[60] Birnbaum Y, Bajaj M, Yang H-C (2018). Combined SGLT2 and DPP4 inhibition reduces the activation of the Nlrp3/ASC inflammasome and attenuates the development of diabetic nephropathy in mice with type 2 diabetes. Cardiovasc Drugs Ther.

[61] Sukhanov S, Higashi Y, Yoshida T (2021). The SGLT2 inhibitor Empagliflozin attenuates interleukin-17A-induced human aortic smooth muscle cell proliferation and migration by targeting TRAF3IP2/ROS/NLRP3/Caspase-1-dependent IL-1β and IL-18 secretion. Cell Signal.

[62] Lyons CL, Roche HM (2018). Nutritional modulation of AMPK-impact upon metabolic-inflammation. Int J Mol Sci.

[63] Cordero MD, Williams MR, Ryffel B (2018). AMP-activated protein kinase regulation of the nlrp3 inflammasome during aging. Trends Endocrinol Metab.

[64] Garcia D, Shaw RJ (2017). AMPK: mechanisms of cellular energy sensing and restoration of metabolic balance. Mol Cell.

[65] Lee H-M, Kim J-J, Kim HJ (2013). Upregulated NLRP3 inflammasome activation in patients with type 2 diabetes. Diabetes.

[66] Hawley SA, Ford RJ, Smith BK (2016). The Na+/glucose cotransporter inhibitor canagliflozin activates AMPK by inhibiting mitochondrial function and increasing cellular AMP levels. Diabetes.

[67] Zhou H, Wang S, Zhu P (2018). Empagliflozin rescues diabetic myocardial microvascular injury via AMPK-mediated inhibition of mitochondrial fission. Redox Biol.

[68] Xu J, Kitada M, Ogura Y (2021). Dapagliflozin restores impaired autophagy and suppresses inflammation in high glucose-treated HK-2 cells. Cells.

[69] Koyani CN, Plastira I, Sourij H (2020). Empagliflozin protects heart from inflammation and energy depletion via AMPK activation. Pharmacol Res.

[70] Li Y, Chen Y (2019). AMPK and autophagy. Adv Exp Med Biol.

[71] He C, Klionsky DJ (2009). Regulation mechanisms and signaling pathways of autophagy. Annu Rev Genet.

[72] Nasiri-Ansari N, Nikolopoulou C, Papoutsi K (2021). Empagliflozin attenuates non-alcoholic fatty liver disease (NAFLD) in high fat diet fed ApoE(-/-) mice by activating autophagy and reducing er stress and apoptosis. Int J Mol Sci.

[73] Jaikumkao K, Promsan S, Thongnak L (2021). Dapagliflozin ameliorates pancreatic injury and activates kidney autophagy by modulating the AMPK/mTOR signaling pathway in obese rats. J Cell Physiol.

[74] DeFronzo RA, Hompesch M, Kasichayanula S (2013). Characterization of renal glucose reabsorption in response to dapagliflozin in healthy subjects and subjects with type 2 diabetes. Diabetes Care.

[75] Song P, Onishi A, Koepsell H (2016). Sodium glucose cotransporter SGLT1 as a therapeutic target in diabetes mellitus. Expert Opin Ther Targets.

[76] Pedersen DJ, Guilherme A, Danai LV (2015). A major role of insulin in promoting obesity-associated adipose tissue inflammation[J]. Molecular Metabolism.

[77] Mauer J, Chaurasia B, Plum L (2010). Myeloid cell-restricted insulin receptor deficiency protects against obesity-induced inflammation and systemic insulin resistance. PLoS Genet.

[78] Aljada A, Ghanim H, Saadeh R (2001). Insulin inhibits NFkappaB and MCP-1 expression in human aortic endothelial cells. J Clin Endocrinol Metab.

[79] La Grotta R, de Candia P, Olivieri F (2022). Anti-inflammatory effect of SGLT-2 inhibitors via uric acid and insulin. Cell Mol Life Sci.

[80] Ekholm E, Hansen L, Johnsson E (2017). Combined treatment with saxagliptin plus dapagliflozin reduces insulin levels by increased insulin clearance and improves β-cell function. Endocr Pract.

[81] Prattichizzo F, De Nigris V, Micheloni S (2018). Increases in circulating levels of ketone bodies and cardiovascular protection with SGLT2 inhibitors: is low-grade inflammation the neglected component?. Diabetes Obes Metab.

[82] Ferrannini E, Mark M, Mayoux E (2016). CV Protection in the EMPA-REG OUTCOME Trial: A 《Thrifty Substrate》 Hypothesis. Diabetes Care.

[83] Ferrannini E, Baldi S, Frascerra S (2016). Shift to fatty substrate utilization in response to sodium-glucose cotransporter 2 inhibition in subjects without diabetes and patients with type 2 diabetes. Diabetes.

[84] Prandi FR, Barone L, Lecis D (2022). Biomolecular mechanisms of cardiorenal protection with sodium-glucose co-transporter 2 inhibitors. Biomolecules.

[85] Youm Y-H, Nguyen KY, Grant RW (2015). The ketone metabolite β-hydroxybutyrate blocks NLRP3 inflammasome-mediated inflammatory disease. Nat Med.

[86] Byrne NJ, Soni S, Takahara S (2020). Chronically elevating circulating ketones can reduce cardiac inflammation and blunt the development of heart failure. Circ Heart Fail.

[87] Nishitani S, Fukuhara A, Shin J (2018). Metabolomic and microarray analyses of adipose tissue of dapagliflozin-treated mice, and effects of 3-hydroxybutyrate on induction of adiponectin in adipocytes. Sci Rep.

[88] Kolb H, Kempf K, Röhling M (2021). Ketone bodies: from enemy to friend and guardian angel. BMC Med.

[89] Vishvanath L, Gupta RK (2019). Contribution of adipogenesis to healthy adipose tissue expansion in obesity. J Clin Investig.

[90] Packer M (2018). Epicardial adipose tissue may mediate deleterious effects of obesity and inflammation on the myocardium. J Am Coll Cardiol.

[91] Kusaka H, Koibuchi N, Hasegawa Y (2016). Empagliflozin lessened cardiac injury and reduced visceral adipocyte hypertrophy in prediabetic rats with metabolic syndrome. Cardiovasc Diabetol.

[92] Patel VB, Shah S, Verma S (2017). Epicardial adipose tissue as a metabolic transducer: role in heart failure and coronary artery disease[J]. Heart Fail Rev.

[93] Sato T, Aizawa Y, Yuasa S (2018). The effect of dapagliflozin treatment on epicardial adipose tissue volume. Cardiovasc Diabetol.

[94] Storgaard H, Gluud LL, Bennett C (2016). Benefits and harms of sodium-glucose co-transporter 2 inhibitors in patients with type 2 diabetes: a systematic review and meta-analysis. PLoS ONE.

[95] Cai X, Yang W, Gao X (2018). The association between the dosage of SGLT2 inhibitor and weight reduction in type 2 diabetes patients: a meta-analysis. Obesity.

[96] Xu L, Ota T (2018). Emerging roles of SGLT2 inhibitors in obesity and insulin resistance: focus on fat browning and macrophage polarization. Adipocyte.

[97] Rosenstock J, Frias J, Páll D (2018). Effect of ertugliflozin on glucose control, body weight, blood pressure and bone density in type 2 diabetes mellitus inadequately controlled on metformin monotherapy (VERTIS MET). Diabetes Obes Metab.

[98] Schork A, Saynisch J, Vosseler A (2019). Effect of SGLT2 inhibitors on body composition, fluid status and renin-angiotensin-aldosterone system in type 2 diabetes: a prospective study using bioimpedance spectroscopy. Cardiovasc Diabetol.

[99] Sakai S, Kaku K, Seino Y (2016). Efficacy and safety of the SGLT2 inhibitor luseogliflozin in Japanese patients with type 2 diabetes mellitus stratified according to baseline body mass index: pooled analysis of data from 52-week phase III trials. Clin Ther.

[100] Bode B, Stenlöf K, Harris S (2015). Long-term efficacy and safety of canagliflozin over 104 weeks in patients aged 55–80 years with type 2 diabetes. Diabetes Obes Metab.

[101] Yabe D, Shiki K, Homma G (2023). Efficacy and safety of the sodium-glucose co-transporter-2 inhibitor empagliflozin in elderly Japanese adults (≥65 years) with type 2 diabetes: a randomized, double-blind, placebo-controlled, 52-week clinical trial (EMPA-ELDERLY). Diabetes Obes Metab.

[102] Zhao Y, Xu L, Tian D (2018). Effects of sodium-glucose co-transporter 2 (SGLT2) inhibitors on serum uric acid level: a meta-analysis of randomized controlled trials. Diabetes Obes Metab.

[103] Braga TT, Forni MF, Correa-Costa M (2017). Soluble uric acid activates the NLRP3 inflammasome. Sci Rep.

[104] So A, Thorens B (2010). Uric acid transport and disease. J Clin Investig.

[105] Ruggiero C, Cherubini A, Ble A (2006). Uric acid and inflammatory markers. Eur Heart J.

[106] Verma S, Ji Q, Bhatt DL (2020). Association between uric acid levels and cardio-renal outcomes and death in patients with type 2 diabetes: a subanalysis of EMPA-REG OUTCOME. Diabetes Obes Metab.

[107] McCormick N, Yokose C, Wei J (2023). Comparative effectiveness of sodium-glucose cotransporter-2 inhibitors for recurrent gout flares and gout-primary emergency department visits and hospitalizations: a general population cohort study. Ann Intern Med.

[108] Yaribeygi H, Atkin SL, Butler AE (2019). Sodium–glucose cotransporter inhibitors and oxidative stress: an update. J Cell Physiol.

[109] Salvatore T, Galiero R, Caturano A (2022). An overview of the cardiorenal protective mechanisms of SGLT2 inhibitors. Int J Mol Sci.

[110] D’Onofrio N, Sardu C, Trotta MC (2021). Sodium-glucose co-transporter2 expression and inflammatory activity in diabetic atherosclerotic plaques: effects of sodium-glucose co-transporter2 inhibitor treatment. Mol Metab.

[111] Hornung V, Bauernfeind F, Halle A (2008). Silica crystals and aluminum salts activate the NALP3 inflammasome through phagosomal destabilization. Nat Immunol.

[112] Halle A, Hornung V, Petzold GC (2008). The NALP3 inflammasome is involved in the innate immune response to amyloid-beta. Nat Immunol.

[113] Wang XX, Levi J, Luo Y (2017). SGLT2 protein expression is increased in human diabetic nephropathy: SGLT2 protein inhibition decreases renal lipid accumulation, inflammation, and the development of nephropathy in diabetic mice. J Biol Chem.

